# IL‐6 expression promoted by Poly(I:C) in cervical cancer cells regulates cytokine expression and recruitment of macrophages

**DOI:** 10.1111/jcmm.14911

**Published:** 2020-01-14

**Authors:** Xin Liu, Lihua Meng, Ling Chen, Ying Liang, Bingyu Wang, Qianqian Shao, Huayang Wang, Xingsheng Yang

**Affiliations:** ^1^ Department of Ultrasound Qilu Hospital of Shandong University Jinan China; ^2^ Department of Gynecology and Obstetrics Qilu Hospital of Shandong University Jinan China; ^3^ Department of Internal Medicine‐Oncology Affiliated Hospital of Shandong Academy of Medical Sciences Shandong First Medical University Jinan China; ^4^ Institute of Basic Medical Sciences Qilu Hospital of Shandong University Jinan China; ^5^ Department of Clinical Laboratory Qilu Hospital of Shandong University Jinan China

**Keywords:** cervical cancer cell, IL‐6, macrophages, poly(I:C), recruitment

## Abstract

Poly(I:C) is a promising adjuvant for cancer treatment vaccines to enhance the host anti‐tumour immune response. However, the roles of poly(I:C) in the cervical cancer microenvironment and local immune reactions are not well understood. In this study, we investigated the roles of poly(I:C) in the cervical cancer. We analysed the cytokine transcription and secretion of cervical cancer cell lines and THP‐1–derived macrophages after poly(I:C) treatment, respectively. These results revealed that IL‐6 was significantly up‐regulated, and this up‐regulation was partly dose dependent. poly(I:C)‐stimulated supernatant of cervical cancer cells promoted M1‐type cytokine IL‐1β and IL‐6 expression of THP‐1–derived macrophages, but inhibited the expression of M2‐type cytokine, IL‐10 and CCL22. The recruitment of THP‐1–derived macrophages by poly(I:C)‐stimulated cervical cancer cell supernatant was also enhanced. Inhibition of IL‐6 expression in cervical cancer cells by siRNA transfection almost completely reversed the effects of poly(I:C) treatment. Finally, we found that phosphorylation of the NF‐κB signalling pathway in cervical cancer cells occurred quickly after poly(I:C) treatment. Moreover, the NF‐κB signalling pathway inhibitor PDTC significantly inhibited poly(I:C)‐induced IL‐6 expression. Taken together, these results suggest that poly(I:C) might regulate the effects of cervical cancer cells on tumour‐infiltrated macrophages, and subsequently promote a pro‐inflammatory tumour microenvironment.

## 1 INTRODUCTION

Cervical cancer is the most common gynaecological malignancy, ranking fourth in both incidence and cancer‐related deaths among women worldwide.^1^ Despite the advancements in prophylactic vaccines and screening programs, which have greatly decreased the incidence rate, effective therapies continue to fall short. A therapeutic vaccine, however, would likely be a promising approach in the treatment of cervical cancer by modulating the host immune system in vivo and thus provoking an anti‐tumour response.

Adjuvants are mandatory in cancer vaccines for their capacity to elicit a strong and persistent immune response towards tumour‐associated antigens, as the immunogenicity of recombinant antigen proteins or peptides alone is usually not sufficient. Polyriboinosinic‐polyribocytidylic acid (poly(I:C)), a synthetic compound that mimics viral dsRNA polymers, is one of the most extensively studied adjuvants of anti‐tumour vaccines.^2^ As the ligand of the Toll‐like receptor 3 (TLR3), poly(I:C) triggers the procession and presentation of antigens in innate immune cells, including macrophages and dendritic cells, subsequently leading to anti‐tumour adaptive immunity.^3^, ^4^

Elevated immunological responses have been observed in various poly(I:C)‐involved strategies over the past decade. In human papillomavirus (HPV)‐infected genital tumours, the application of poly(I:C), along with HPV16E7 peptide vaccination, has increased, by approximately fivefold, the number of vaccine‐specific CD8+ T cells infiltrated in the genital mucosa of mice.^5^ This observation is particularly interesting, considering that the levels of inflammatory cytokine expression and immunocyte infiltration were usually low in high‐risk HPV‐infected lesions, especially in cervical cancer, due to the immunosuppression effect of HPV oncoproteins.^6^, ^7^, ^8^ One possible explanation was that poly(I:C) modulated the tumour microenvironment, an established mechanism lacking detailed investigation.

The tumour microenvironment contains various myeloid lineage cells that usually show unique tumour‐supportive characteristics compared with their companion residents in normal tissue. In cancer vaccination, tumour‐derived nucleic acids, such as poly(I:C), act on both tumour cells and non‐malignant components, resulting in a complex interaction network. Previous research has shown that the cytosolic effect of poly(I:C) evokes an inflammatory form of death in tumour cells, as well as several other effects including type I IFN induction and myeloid cell maturation and activation.^9^ In normal keratinocytes, which are host cells of HPV, extracellular poly(I:C) stimulation greatly induced inflammatory mediator expression, including tumour necrosis factor α and type I IFNs, therefore promoting the activation of dendritic cells.^10^ Similar observations were also achieved in cervical cancer cells in which poly(I:C) enhanced the expression of necroptosis regulator RIPK3, and increased IL‐12 production in dendritic cells.^11^ However, the effects of poly(I:C) on the interactions between the tumour microenvironment and tumour‐infiltrated macrophages, the number of which was almost ten times higher than that of dendritic cells,^12^ have not been fully explored.

In this study, we demonstrate that cervical cancer cells stimulated by poly(I:C) regulate cytokine expression and recruitment of THP‐1–derived macrophages. The enhanced IL‐6 expression in cervical cancer cells through the NF‐κB signalling pathway was shown as a critical factor in the modulation of poly(I:C)‐stimulated cervical cancer cells on macrophages.

## 2 MATERIALS AND METHODS

### 2.1 Cell culture and generation of THP‐1–derived macrophages

Human cervical cancer cell lines HeLa and CaSki and acute monocytic leukaemia cell line THP‐1 were obtained from the American Type Culture Collection (ATCC). The cells were cultured in RPMI‐1640 medium supplemented with heat‐incubated 10% FBS, 100 U/mL penicillin and 100 μg/mL streptomycin at 37°C in an incubator with 5% CO_2_.

To obtain the conditioned medium (CM) of HeLa and CaSki cells, 2 × 10^5^ HeLa and CaSki cells were seeded into 2 mL RPMI‐1640 medium/10% FBS into a 6‐well plate. After 6 hours, different concentrations of poly(I:C) were added to the medium. The medium was then washed and replaced with new RPMI‐1640 medium with 10% FBS for another 12 hours. Cell supernatant was collected as HeLa and CaSki CM for later experiments.

THP‐1–derived macrophages were generated as previously described.^13^ Precisely, 1 × 10^6^ THP‐1 cells were treated with 100 ng/mL PMA for 48 hours and adherent cells were collected for later experiments. In some cases, the medium was replaced with 50% total volume of the CM of cervical cancer cells after 6 hours of treatment with phorbol myristate acetate (PMA), and THP‐1 cells were further cultured for another 42 hours with the concentration of PMA maintained at 100 ng/mL.

### 2.2 Enzyme‐linked immunosorbent assay (ELISA)

The concentrations of IL‐1β, IL‐2, IL‐4, IL‐6, IL‐8, IL‐10, IFN‐γ, MCP‐1, TNF‐α and CCL22 in CM of HeLa, CaSki or THP‐1–derived macrophages were determined by ELISA kits (R&D Systems) according to the manufacturer's instructions.

### 2.3 Real‐time quantitative RT‐PCR

Total RNA was extracted using TRIzol reagent (Invitrogen), and the cDNA was synthesized by reverse transcription. Quantitative RT‐PCR was performed on a LightCycler 2.0 (Roche Diagnostic). GAPDH was used as an internal control. The mRNA level of each sample was measured by the 2−ΔΔT method. The primer sequences were listed in the supplementary documents (Table S1).

### 2.4 Small interfering RNA transfection

Small interfering RNA (siRNA) targeting human IL‐6 and a scramble‐negative control was designed by GeneChem Co., Ltd. For transient silencing, 5 × 10^5^ of HeLa cells were seeded into 200 μL serum‐free RPMI‐1640 medium in 24‐well plates to reach 60% confluence. A 100 μL of mixture of Lipofectamine 2000 (Invitrogen) and IL‐6 siRNA or negative control siRNA was added into the well for 6 hours. Each well was refilled with 200 μL complete medium (containing 10% foetal bovine serum [FBS]) for another 48 hours. In some cases, 25 μg/mL of poly(I:C) was added into the well at the 36‐hour time‐point and the experiment proceeded for 12 more hours to match the treatment period of non‐transfection groups. The medium was then replaced with 1 mL new RPMI‐1640 medium with 10% FBS for 12 hours, after which the supernatant was collected. The sequences of human IL‐6 and scramble siRNA were 5′‐CUUCCAAUCUGGAUUCAAU‐3′ and 5′‐GGGCAAGACGAGCGGGAAG‐3′, respectively.

### 2.5 Migration assay

A total of 1 × 10^5^ THP‐1–derived macrophages were resuspended in 100 μL of serum‐free RPMI‐1640 medium and added into the upper compartment of the Transwell inserts (24‐well plate, 8‐μm pores, BD Biosciences). RPMI‐1640 medium (600 μL) containing 20% FBS, or the CM of cervical cancer cells, was added into the lower chamber of the plate. After incubating at 37°C in 5% CO_2_ for 24 hours, the migrated cells on the lower surface of the filter were washed and fixed by 10% formalin and stained with eosin. Five random fields of each well were photographed, and cell numbers were counted.

### 2.6 Western blot

Cells in six‐well plates were washed with PBS and lysed with equal volumes of RIPA lysis buffer containing 1 mmol/L phenylmethylsulfonyl fluoride (PMSF) on ice and then centrifuged for 10 minutes at 300 *g* under 4°C. Lysates with equal amounts of protein were separated by 10% SDS‐PAGE and then transferred onto a polyvinylidene fluoride (PVDF) membrane (Millipore). The membranes were blocked for 1 hour at room temperature with 5% BSA in TBS containing 0.1% Tween‐20 and then incubated overnight at 4°C with NF‐κB (Cell Signaling Technology) or β‐actin (Santa Cruz Biotechnology) antibody. The membranes were exposed to horseradish peroxidase‐labelled secondary antibodies (1:3000) for 1 hour at room temperature and detected by enhanced chemiluminescence detection systems (Amersham Imager 600, GE Healthcare Life Sciences, and ChemiDoc™ Touch Imaging System, Bio‐Rad).

### 2.7 Statistical analysis

All experiments were performed three times. The statistical analyses were performed, and experimental graphs were generated using SPSS 17.0 and GraphPad Prism software, respectively. Descriptive statistics, including the mean ± SD and paired/unpaired Student's *t* test and one‐way ANOVA tests, were used to analyse the significance of differences. *P* values of <.05 were considered significant (*P < .05, **P < .01).

## 3 RESULTS

### 3.1 Poly(I:C) promotes the secretion of IL‐6 in cervical cancer cell lines

**Figure 1:**
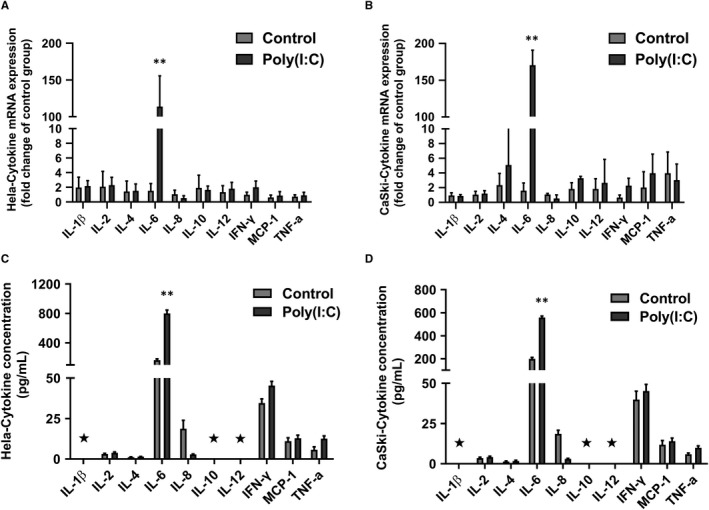
Poly(I:C) treatment promoted IL‐6 secretion in cervical cancer cells. The cytokine mRNA levels of HeLa (A) and CaSki (B) cells in control of poly(I:C) (25 μg/mL)‐treated groups were tested by qRT‐PCR. The cytokine expression of HeLa (C) and CaSki (D) conditioned medium in the control or poly(I:C) (25 μg/mL)‐treated groups (12 h) was tested by ELISA. The mRNA or protein level of each cytokine in poly(I:C)‐treated group was compared with the corresponding control group. Each bar represents mean ± SD (n = 3. *P < .05; **P < .01. ⋆, below minimum detection limit)

To explore the effect of poly(I:C) on the secretory activity of cervical cancer cells, two cervical cancer cell lines, HeLa and Caski, were treated by poly(I:C) (25 μg/mL). The levels of cytokines in the supernatants of the two cervical cancer cell lines were determined by qRT‐PCR and ELISA after 12 hours of poly(I:C) treatment. qRT‐PCR results showed that IL‐6 mRNA transcription was significantly up‐regulated after poly(I:C) treatment in both two cervical cell lines (Figure 1A,B). Results showed that the expression profiles of cytokines detected in the two cervical cancer cell lines were consistent and IL‐2, IL‐4, IL‐6, IL‐8, IFN‐γ, MCP‐1 and TNF‐α were all expressed. The expression level of IL‐6 was considered to be high (approximately 200 pg/mL), and the level of IL‐1β, IL‐10 and IL‐12 in conditioned medium was below the minimum detection limit. After poly(I:C) treatment, IL‐6 expression levels were significantly up‐regulated in both cell lines, and HeLa cells had higher IL‐6 expression than CaSki cells (Figure 1C,D).

**Figure 2:**
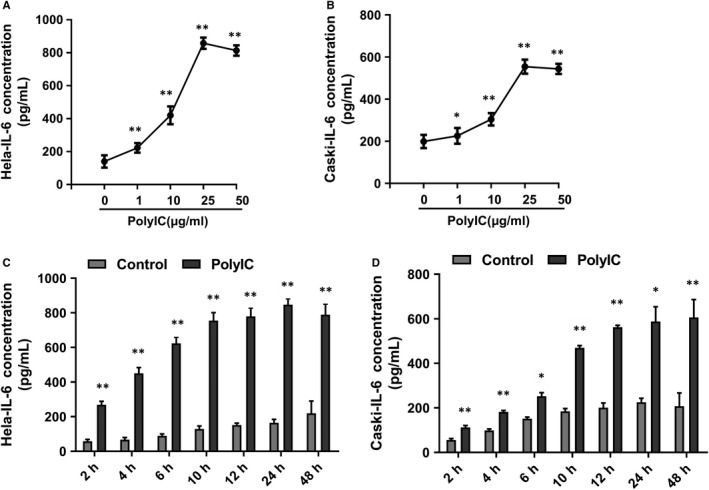
The secretion of IL‐6 with different concentrations or times of poly(I:C) treatment. The level of IL‐6 in HeLa (A) or CaSki (B) supernatant with different concentrations of poly(I:C) treatment for 12 hours was analysed by ELISA. The level of IL‐6 in HeLa (C) or CaSki (D) supernatant with 25 μg/mL of poly(I:C) treatment for intended period of time was also analysed. IL‐6 secretion level in poly(I:C)‐treated group for intended period of time was compared with corresponding control group of the same time‐point. Each bar represents mean ± SD (n = 3. *P < .05; **P < .01.)

We further explored whether the regulation of poly(I:C) on IL‐6 expression in cervical cancer is related to its concentration and duration of action. As shown in Figure 2A,B, poly(I:C) dose dependently promoted IL‐6 expression in HeLa and CaSki cells, with the most significant effect at 25 μg/mL. The up‐regulation of IL‐6 secretion was found after 2 hours of poly(I:C) treatment in both two cervical cancer cell lines. In HeLa cells, the level of IL‐6 in conditioned medium became relatively stable after 10 hours of poly(I:C) treatment. The secretion of IL‐6 in CaSki cells was continuously elevated until 12 hours of poly(I:C) treatment (Figure 2C,D).

### 3.2 Poly(I:C) regulates the effect of cervical cancer cells on the secretion of cytokines from THP‐1–derived macrophages

**Figure 3:**
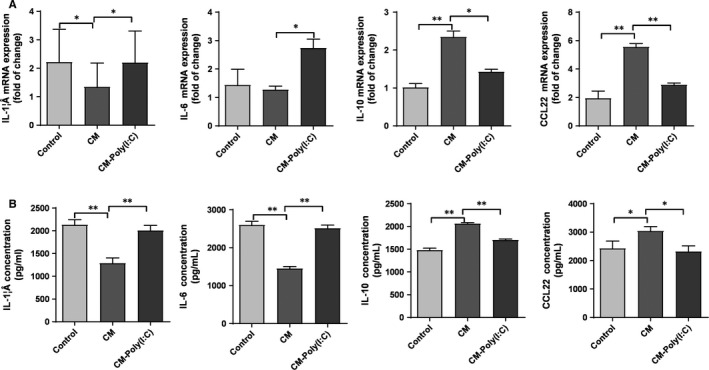
Cytokine secretion profiles of THP‐1–derived macrophages were regulated by CM of poly(I:C)‐treated cervical cancer cells. THP‐1–derived macrophages were treated with RPMI‐1640 (control), CM of HeLa cells (CM) or CM of HeLa cells stimulated by 25 μg/mL of poly(I:C) for 12 h (CM‐poly(I:C)). mRNA level (A) and supernatant concentration (B) of IL‐1β, IL‐6, IL‐10 and CCL22 were measured. The RT‐PCR data were normalized to the control and shown as fold change. Each bar represents mean ± SD (n = 3. *P < .05; **P < .01.)

To investigate the effect of poly(I:C)‐treated cervical cancer cells on macrophage cytokine expression profiles, we collected conditioned medium (CM) with or without poly(I:C) treatment. The CM was added during the inducing differentiation of the THP‐1 monocyte line into macrophages by PMA. The treatment procedure was described in the Methods section above. As shown in Figure 3, HeLa cell CM without poly(I:C) inhibited the expression of pro‐inflammatory cytokines IL‐1β and IL‐6 in THP‐1–derived macrophages, while the transcription and secretion levels of IL‐10 and CCL22 were significantly higher. In contrast, 12 hours of poly(I:C) treatment almost completely reversed the regulatory effect of HeLa CM on the secretion profile of THP‐1–derived macrophages.

### 3.3 Poly(I:C)‐promoted IL‐6 expression is involved in the regulation of cervical cancer cells on the secretion profile of THP‐1–derived macrophages

**Figure 4:**
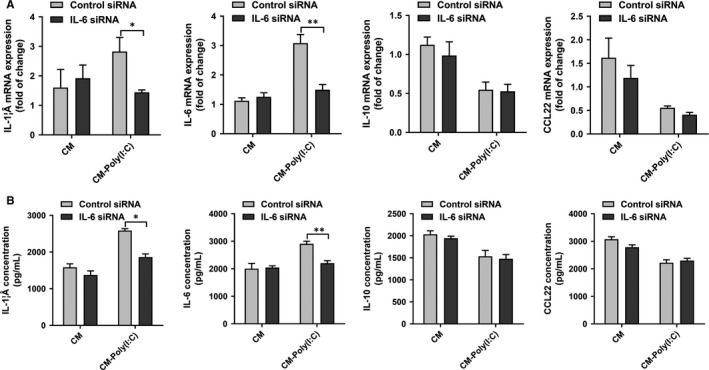
IL‐6 was involved in the regulation of cytokine profile of THP‐1–derived macrophages by poly(I:C)‐treated cervical cancer cells. THP‐1–derived macrophages were treated with CM of cervical cancer cells, which was pre‐treated by control or IL‐6 siRNA, with/without poly(I:C) stimulation. mRNA level (A) and supernatant concentration (B) of IL‐1β, IL‐6, IL‐10 and CCL22 were measured. The RT‐PCR data were normalized to the control and shown as fold change. Each bar represents mean ± SD (n = 3. *P < .05; **P < .01.)

Since poly(I:C) was found to promote the expression of IL‐6 in cervical cancer cells, we further explored whether IL‐6 is involved in the regulation of macrophage secretion by cervical cancer. First, the HeLa cell line was transfected with control or IL‐6 siRNA to inhibit the expression of IL‐6. After transfection for 36 hours, poly(I:C) (25 μg/mL) was added, and the CM was collected after culturing for 12 hours. The CM was then added during the process in which THP‐1 induced differentiation into macrophages. The level of transcription and secretion of the cytokines were detected after complete differentiation into THP‐1–derived macrophages. As shown in Figure 4, cervical cancer cell CM transfected with control siRNA or IL‐6 siRNA showed no significant difference in regulation of cytokine expression of THP‐1–derived macrophages when there was no poly(I:C) effect. IL‐6 silencing in cervical cancer cells significantly inhibited the transcription and secretion of pro‐inflammatory cytokines IL‐1β and IL‐6 by THP‐1–derived macrophages, but had no significant effect on the expression of IL‐10 and CCL22.

### 3.4 Poly(I:C) promotes recruitment of THP‐1–derived macrophages by cervical cancer cells

**Figure 5:**
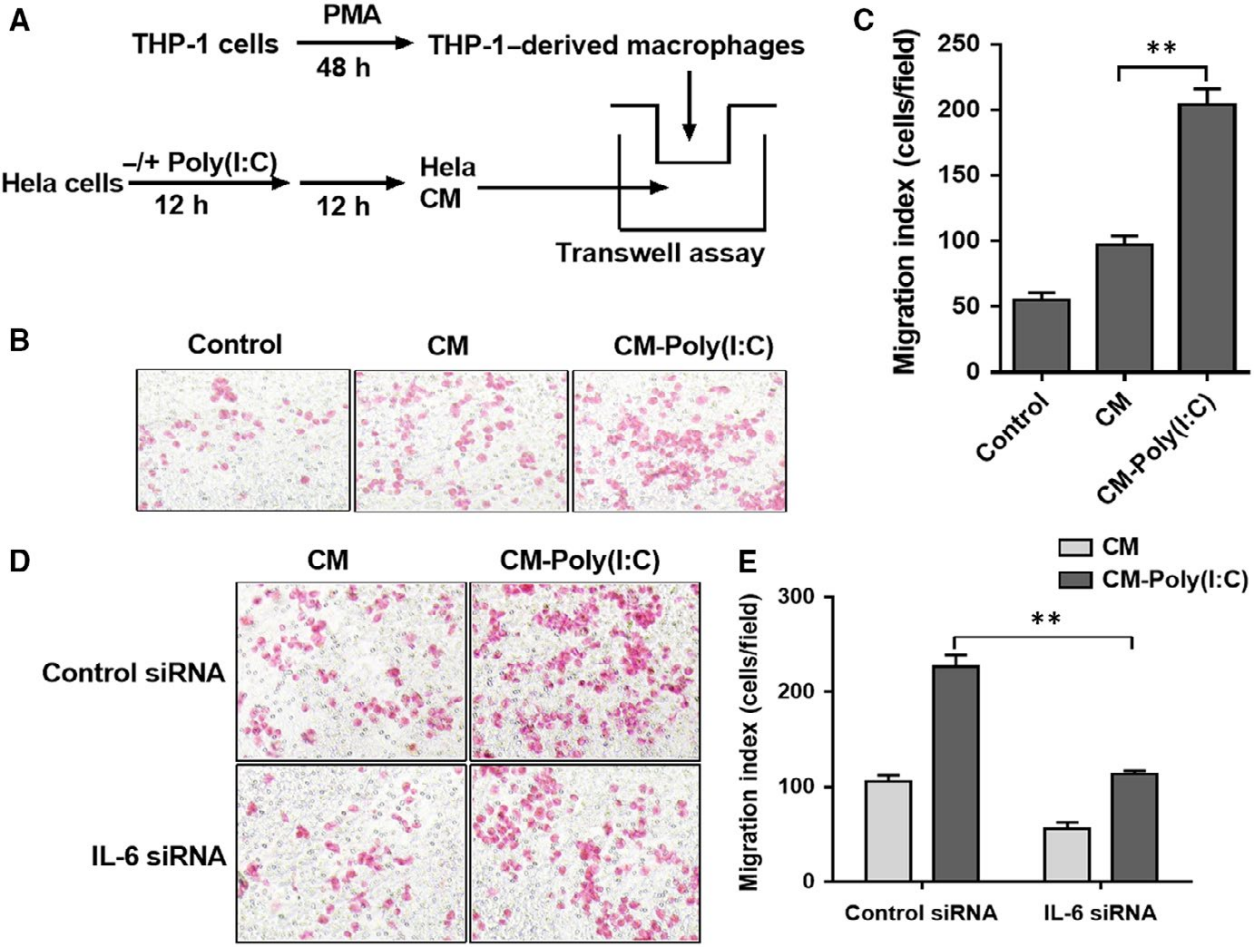
IL‐6 was involved in the recruitment of THP‐1–derived macrophages by poly(I:C)‐treated cervical cancer cells. (A) The diagrammatic illustration for the Transwell assay procedure. (B) A typical field of THP‐1–derived macrophages recruited by RPMI‐1640 (control), CM of HeLa cells (CM) or CM of HeLa cells stimulated by 25 μg/mL of poly(I:C) for 12 h (CM‐poly(I:C)). (C) The number of recruited THP‐1–derived macrophages in three groups. (D) A typical field of THP‐1–derived macrophages recruited by CM of HeLa cells, pre‐treated by control or IL‐6 siRNA with/without poly(I:C) stimulation. (E) The number of recruited THP‐1–derived macrophages in four groups. Each bar represents mean ± SD (n = 3. **P < .01.)

To investigate whether poly(I:C) treatment affects the recruitment of macrophages by cervical cancer cells, we used Transwell chambers to establish an in vitro cell migration model. As shown in Figure 5A, THP‐1–derived macrophages were placed in the upper compartment of the chamber, and the lower chamber was filled with cervical cancer cell CM with/without poly(I:C) action. The number of migrated THP‐1–derived macrophages was observed after 24 hours. The results showed that cervical cancer cell CM with poly(I:C) function significantly promoted the migration of THP‐1–derived macrophages (Figure 5B,C).

We further explored the role of IL‐6 in the promotion of macrophage recruitment by cervical cancer cells in poly(I:C). (Figure 5D,E). The recruitment of HeLa cell CM transfected with IL‐6 siRNA to THP‐1–derived macrophages was significantly inhibited compared with control siRNA.

### 3.5 Poly(I:C) promotes the expression of IL‐6 in cervical cancer cells via the NF‐κB pathway

**Figure 6:**
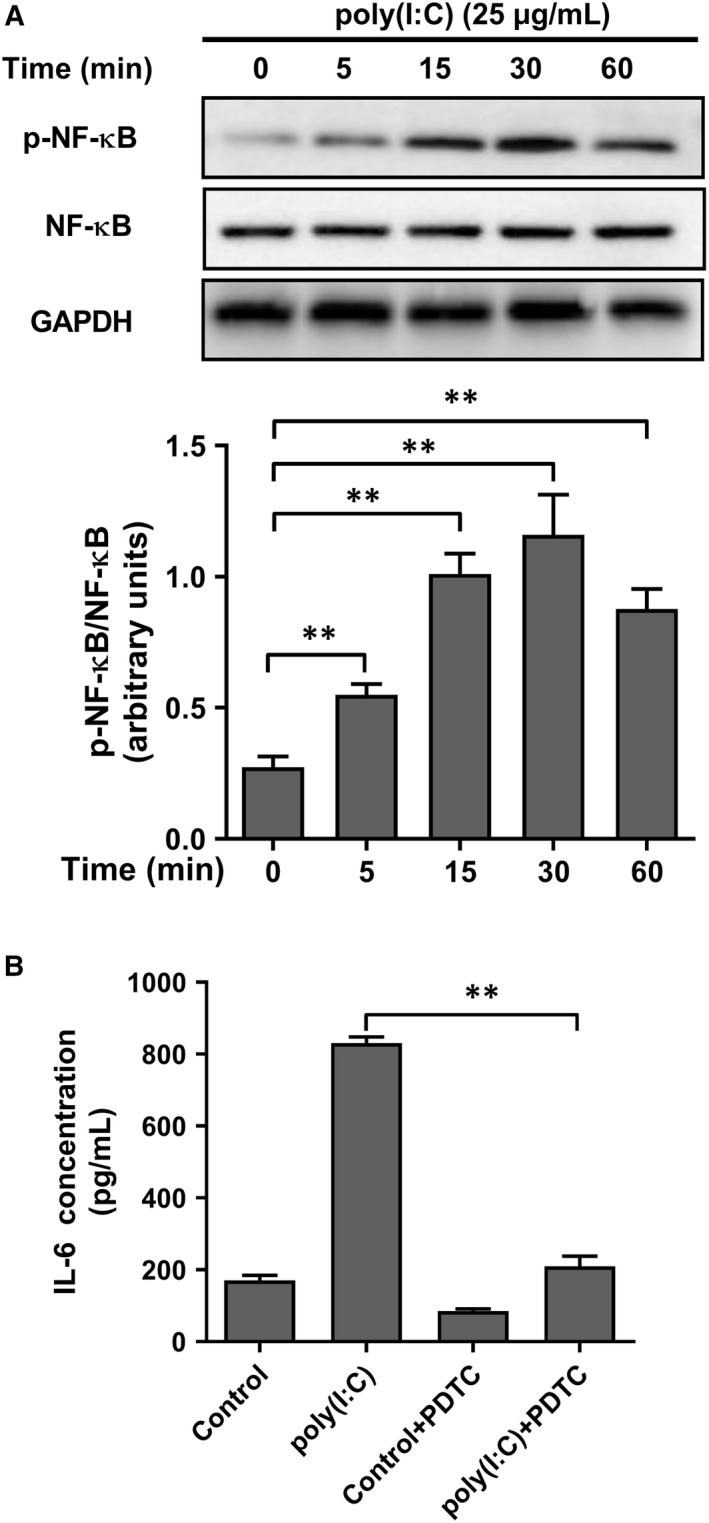
Poly(I:C)‐promoted IL‐6 expression was depend on activation of NF‐κB signalling pathway. (A) Phosphorylation of NF‐κB (pNF‐κB) and total NF‐κB was measured at different time‐points after poly(I:C) stimulation (upper), and the ratio of pNF‐κB and total NF‐κB was calculated (lower). GAPDH was analysed as loading control. (B). The IL‐6 concentration of HeLa cells pre‐treated by NF‐κB inhibitor, PDTC, with/without poly(I:C) stimulation. Each bar represents mean ± SD (n = 3. **P < .01.)

NF‐κB is a signalling pathway closely related to inflammatory reactions. In this study, we found that the level of phosphorylated NF‐κB in HeLa cells was up‐regulated 5 minutes after poly(I:C) treatment, reaching a peak at 30 minutes, suggesting that the signalling pathway is activated (Figure 6A). To investigate whether the activated NF‐κB signalling pathway is involved in poly(I:C)‐regulated IL‐6 expression, we selected the common NF‐κB signalling pathway inhibitor PDTC (4 μg/mL) to pre‐treat HeLa cells for 1 hour, then changed the medium and added poly(I:C). ELISA results showed that PDTC significantly reversed the effect of poly(I:C) on IL‐6 secretion in HeLa cells (Figure 6B).

## 4 DISCUSSION

Poly(I:C) is an important adjuvant component in therapeutic tumour vaccines and can promote the body's anti‐tumour immunity. However, little is known about whether the topical application of poly(I:C) alters the local microenvironment of the tumour, thereby affecting the functional activity of the locally infiltrating immune cells. In this study, we explored the role of poly(I:C) in HPV‐positive cervical cancer cells and found that poly(I:C) promotes the expression of IL‐6 in cervical cancer cells by activating the NF‐κB signalling pathway. Increased secretion of IL‐6 changes the regulation of cervical cancer cells on the secretion profile of macrophage cytokines and promotes the recruitment of macrophages in cervical cancer cell supernatants. These results suggest that poly(I:C) is involved in the regulation between tumour cells and tumour‐infiltrating macrophages in the local microenvironment of cervical cancer.

Cervical cancer cells express TLR3 and are therefore one of the target cells of poly(I:C).^14^ We examined the cytokine expression profiles of two different HPV‐positive cervical cancer cells, HeLa (HPV18+) and Caski (HPV16+), and found that both cervical cancer cells highly expressed IL‐6, which is consistent with previous studies.^15^ Moreover, after the poly(I:C) treatment, the expression of IL‐6 in these two cervical cancer cell lines was rapidly up‐regulated within 12 hours of poly(I:C) treatment and remained stable for 48 hours.

IL‐6 is a characteristic cytokine in cervical cancer, which is up‐regulated in CIN III and during the tumour stage.^16^ High expression of IL‐6 in serum or tumour is an unfavourable factor in the prognosis of patients with cervical cancer.^17^ As cervical cancer cells themselves have no IL‐6 receptor α‐chain,^18^, ^19^ this pleiotropic cytokine might regulate the non‐tumour cell components through paracrine action, mainly local‐infiltrated immune cells, and be involved in the establishment of a tumour‐supportive microenvironment.

Due to the immunosuppressive effects of HPV, the local anti‐tumour immune activity of cervical cancer is usually defective. In the low‐grade intraepithelial neoplasia (CIN I‐II) stage with HPV infection, the expression of pro‐inflammatory cytokines and the number of infiltrated inflammatory cells are significantly reduced,^20^, ^21^ which is an important factor to promote further malignant transformation of low‐grade precancerous lesions. Despite the increased number of immune cells infiltrating in the subsequent high‐grade intraepithelial neoplasia (CIN III) to cervical cancer, the tumour is still immunosuppressed and regulates the phenotype and function of locally infiltrating immune cells.^22^, ^23^ Tumour‐infiltrating macrophages usually migrate from peripheral blood mononuclear cells and localize and differentiate in the tumour microenvironment. Therefore, they are more susceptible to local environment regulation than adaptive immune cells. Previous studies^24^ have shown that IL‐10 expression was significantly up‐regulated in macrophages infiltrating in high‐grade cervical intraepithelial neoplasia (CIN II‐III) and cervical cancer tissue, and this up‐regulation was more prominent in cases with high‐risk HPV infection and high viral load.

In the present study, we used an in vitro model to mimic the effects of cervical cancer cell secretion on macrophage differentiation. The results showed that the addition of cervical cancer cell supernatant during the differentiation of monocytes into macrophages inhibited the expression of M1 cytokines such as IL‐1β and IL‐6 and promoted M2 cytokines such as IL‐10 and CCL22 expression. This is consistent with the phenomenon observed in tumour tissue. After poly(I:C) treatment, the regulation of the secretion spectrum of macrophages by cervical cancer cells was reversed. Another in vitro experiment^25^ showed that cervical cancer cell culture supernatant can induce THP‐1–derived macrophages to exhibit a stable M2‐like phenotype of CD163^+^ CD206^+^ and increase IL‐6 expression. However, the M2 type cytokines IL‐4 and IL‐10 were not significantly changed, which is different from our findings. This difference may be related to the timing of the addition of cervical cancer cell supernatant, given that, instead of adding the cervical cancer cell supernatant into macrophages after 6 hours of PMA treatment in our study, the previous study added the cervical cancer supernatant after 3 days of PMA treatment. It is believed that THP‐1 has differentiated into mature macrophages (M0) after 3 days. Therefore, our study focused more on the effects of cervical cancer on the later status of macrophages, in other words, polarization. Results suggest that the same intervention condition affects different stages of macrophage differentiation, and may have an impact on the functional activity of macrophages, and even lead to opposite results.

The promotion of IL‐1β and IL‐6 expression of macrophages by poly(I:C)‐treated supernatants was reversed by IL‐6 siRNA interfering in cervical cancer cells. In addition, the recruitment of the supernatant to macrophages is also inhibited. These two observations suggested that the involvement of poly(I:C) promoted IL‐6 secretion of cervical cancer in the regulation of cellular behaviours of macrophages. Previous studies have also shown that IL‐6 is involved in the accumulation of local macrophages in cervical cancer.^17^, ^23^, ^26^ Moreover, it has been demonstrated that HPV‐transformed cervical cancer cells induce accumulation of monocytes in tumours through a CCL2‐CCR2–positive feedback loop activated by IL‐6 and M‐CSF, and further promote tumour invasion by enhancing MMP‐9 expression on monocytes.^27^ In addition, cervical cancer‐derived IL‐6 promotes the expression of CCL20 in cervical fibroblasts, which further promotes the recruitment of tumour‐promoting Th17 cells to the tumour site.^28^ The above results suggest that IL‐6 is one of the key molecules connecting local chronic inflammatory response and tumour development.^29^ Our results show that poly(I:C) up‐regulated cervical cancer cell‐derived IL‐6 not only promotes the migration of macrophages to tumours, but also promotes the secretion of IL‐6 in macrophages, thus forming a positive feedback loop. This pathway may be one of the important factors for the formation of a special feature of local high IL‐6 expression in cervical cancer. This mechanism of action can further amplify the local effects of IL‐6 in cervical cancer, including intratumoral anti‐tumour immune activity and regulation of other non‐tumour cell components, which ultimately constitute a special microenvironment that promotes tumour development.

Compared with other pattern recognition receptors (PRRs) such as TLR9, the distribution of poly(I:C) receptor TLR3 is not limited to antigen‐presenting cells, but is also distributed in a large number of epidermal fibroblasts, keratinocytes and muscle cells.^2^, ^30^ Therefore, poly(I:C) has a prominent advantage in topical application in superficial parts of the human body, such as subcutaneous injection, intramuscular injection or intravaginal application. However, it should be noted that the wide distribution of TLR3 might cause a significant increase in the risk of excessive activation of the inflammation response by poly(I:C) as an adjuvant. Chronic inflammatory response has been one of the most serious adverse reactions of poly(I:C) injection,^2^ but the mechanisms responsible for this adverse reaction are poorly understood. Our results suggest that poly(I:C) can promote the expression of IL‐6 in cervical cancer cells, induce the local recruitment of macrophages and the secretion of pro‐inflammatory cytokines, while the resulting inflammatory environment increases the risk of tumour progression. However, it is unknown whether the phenomenon observed in in vitro experiments can reflect the in vivo situation, because poly(I:C) is hydrolysed in a short time in serum with a half‐life of only about 30 minutes.^31^

Nevertheless, our observations suggest that when poly(I:C) is selected as a therapeutic vaccine for cervical cancer, special attention should be paid to its effect on the local microenvironment of the tumour, and relevant measures should be taken to inhibit its promotion of IL‐6 secretion. One possible measure is to reduce the application dose of poly(I:C). Observations in animal models showed that intraperitoneal injection of poly(I:C) at a dose of 750 μg/Kg caused fever and a transient increase in plasma IL‐6 and TNF‐α.^32^ Another study showed that the severity of insulitis in diabetes‐prone BioBreeding (BB) rats caused by poly(I:C) was dose‐dependent; however, low‐dose poly(I:C) (0.05 mg/g body weight) was shown to be effective in preventing the transcription of TNF‐α in splenocytes and the development of insulitis.^33^ In this study, we noted that there is a correlation between the expression level of IL‐6 in cervical cancer cells and poly(I:C) concentration. When poly(I:C) concentration was 1 μg/mL, IL‐6 expression was significantly promoted, but the secretion level was significantly lower than that of the 25 μg/mL poly(I:C) treatment group. This suggests that the dose of poly(I:C) should be carefully selected before application in tumour treatment to maintain its immune activation characteristics while avoiding its potential risk of promoting tumour development.

In conclusion, we found that poly(I:C) promoted the secretion of IL‐6 by cervical cancer cells through activation of the NF‐κB pathway. The elevated IL‐6 level in cervical cancer supernatant regulated the secretion profile of THP‐1–derived macrophages and promoted the recruitment of macrophages by cervical cancer supernatant. This finding suggests that poly(I:C) as an adjuvant may enhance the local inflammatory response in cervical cancer, but also could be a risk in cervical cancer immunotherapy. Measures such as appropriate application doses should be taken into consideration to control its effects on non‐immune cells and reduce the risk of tumour development. Despite this, there is still much to be determined in our research. For example, does poly(I:C) affect the anti‐tumour activity of local‐infiltrated macrophages by IL‐6? Does poly(I:C) have a similar affect in other types of tumours? In future research, we will use a tumour‐bearing animal model to explore the regulation of poly(I:C) on local immune composition and functional activity of cervical cancer and related mechanisms.

## CONFLICT OF INTEREST

All the authors declare no conflict of interest.

## AUTHORS' CONTRIBUTION

XL, HW and XY designed these experiments. XL, QS, BW, XY and LC involved in performing the experiment and analysed the data. XL and LM contributed to manuscript preparation and wrote the manuscript. XL and XY revised the manuscript.

## Data Availability

All data used to support the findings of this study are available from the corresponding author upon request.
